# Early dynamic changes to monocytes following major surgery are associated with subsequent infections

**DOI:** 10.3389/fimmu.2024.1352556

**Published:** 2024-04-09

**Authors:** Timothy Arthur Chandos Snow, Alessia V. Waller, Richard Loye, Francis Ryckaert, Antonio Cesar, Naveed Saleem, Rudra Roy, John Whittle, Ahmed Al-Hindawi, Abhishek Das, Mervyn Singer, David Brealey, Nishkantha Arulkumaran, the University College London Hospitals Critical Care Research Team

**Affiliations:** ^1^ Bloomsbury Institute of Intensive Care Medicine, University College London, London, United Kingdom; ^2^ Centre for Anaesthesia, Critical Care & Pain Medicine, University College London, London, United Kingdom; ^3^ Division of Infection & Immunity, University College London, London, United Kingdom; ^4^ National Institute for Health and Care Research, University College London Hospitals Biomedical Research Centre, University College London Hospitals, London, United Kingdom

**Keywords:** immune function, intensive care unit, lymphocyte, monocyte, post-operative infection

## Abstract

**Background:**

Post-operative infections are a common cause of morbidity following major surgery. Little is understood about how major surgery perturbs immune function leading to heightened risk of subsequent infection. Through analysis of paired blood samples obtained immediately before and 24 h following surgery, we evaluated changes in circulating immune cell phenotype and function across the first 24 h, to identify early immune changes associated with subsequent infection.

**Methods:**

We conducted a prospective observational study of adult patients undergoing major elective gastrointestinal, gynecological, or maxillofacial surgery requiring planned admission to the post-anesthetic care unit. Patients were followed up to hospital discharge or death. Outcome data collected included mortality, length of stay, unplanned intensive care unit admission, and post-operative infections (using the standardized endpoints in perioperative medicine–core outcome measures for perioperative and anesthetic care criteria). Peripheral blood mononuclear cells were isolated prior to and 24 h following surgery from which cellular immune traits including activation and functional status were assessed by multi-parameter flow cytometry and serum immune analytes compared by enzyme-linked immunosorbent assay (ELISA).

**Results:**

Forty-eight patients were recruited, 26 (54%) of whom developed a post-operative infection. We observed reduced baseline pre- and post-operative monocyte CXCR4 and CD80 expression (chemokine receptors and co-stimulation markers, respectively) in patients who subsequently developed an infection as well as a profound and selective post-operative increase in CD4^+^ lymphocyte IL-7 receptor expression in the infection group only. Higher post-operative monocyte count was significantly associated with the development of post-operative infection (false discovery rate < 1%; adjusted p-value = 0.001) with an area under the receiver operating characteristic curve of 0.84 (p < 0.0001).

**Conclusion:**

Lower monocyte chemotaxis markers, higher post-operative circulating monocyte counts, and reduced co-stimulatory signals are associated with subsequent post-operative infections. Identifying the underlying mechanisms and therapeutics to reverse defects in immune cell function requires further exploration.

## Introduction

Post-operative infections are a significant cause of morbidity, affecting up to 40% of patients undergoing major surgery (1, 2). Surgery activates the immune system in response to physical damage to tissues (“sterile inflammation”), with many similarities with infections (3). This response is influenced by a myriad of factors including patient age, medical conditions (e.g., cancer), and medications. A number of immune responses following surgery are associated with subsequent infections (4, 5). Two well-characterized changes associated with post-operative infections are a reduction in monocyte HLA-DR (human leukocyte antigen–DR isotype) and persistent lymphopenia (6, 7). Similar changes are seen in patients who die from sepsis (8).

Despite these known associations, the duration, intensity, and characteristics of the immune response to surgery and its impact on the response to infections remain poorly characterized. Better understanding may facilitate identification of high-risk patients, the risk period, and preventative therapies.

We hypothesized that immune pathways that are reproducibly perturbed early after major surgery may guide approaches to modulate immune responses to mitigate the risk of subsequent infections. We therefore evaluated if there were differences in changes to immune cell phenotype before and 24 h following surgery between patients who did and did not develop a post-operative infection. We also investigated the *in vitro* immune response to an infectious challenge before and after surgery, to determine if surgery altered the immune response to a subsequent infectious challenge, and if this was different between patients who did and did not develop a post-operative infection.

**Figure 1:**
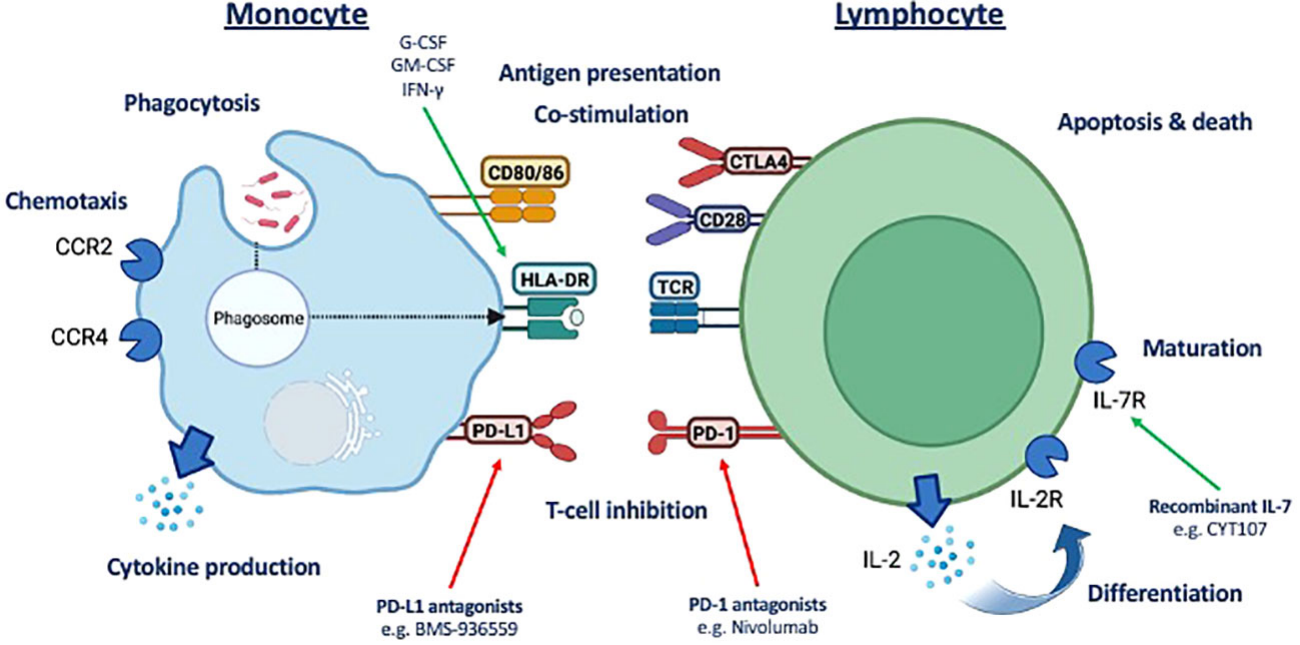
Summary of potential druggable monocyte and T-lymphocyte immune pathway targets assessed. Assessment of a panel of druggable immune targets and associated pathways typically associated with immunosuppression in sepsis underpinning the commonality in the immune response to surgery and sepsis. CD, cluster of differentiation; HLA-DR, human leukocyte antigen–DR isotype; LD, live/dead; CCR2, C-C motif chemokine receptor 2; CXCR4, CXC motif chemokine receptor 4; IL, interleukin; CTLA-4, cytotoxic T-lymphocyte–associated protein 4; TCR, T-cell receptor, PD-1, programmed cell death protein-1; PD-L1, programmed death-ligand 1.

The commonality in the immune response to surgery and sepsis focused our analysis to a panel of druggable immune targets and associated pathways typically associated with immunosuppression in sepsis (3, 8, 9). This included receptors commonly associated with monocyte antigen presentation and co-stimulation (HLA-DR, CD80, and CD86), immune checkpoint inhibition [lymphocyte PD-1 (programmed cell death protein-1) and monocyte PD-L1 (programmed death-ligand 1)], lymphocyte proliferation/maturation [interleukin-2 (IL-2) and IL-7 receptors], lymphocyte activation [CD28 and cytotoxic T-lymphocyte–associated protein 4 (CTLA-4)], and lymphocyte viability. We also assessed monocyte chemokine receptors (CCR2 and CXCR4) and intracellular cytokines for a more comprehensive analysis (**Figure 1**).

## Materials and methods

### Ethics

Ethical approval for obtaining clinical samples and data was received from the London – Queen Square Research Ethics Committee (REC reference 20/LO/1024). Ethics for obtaining healthy volunteer samples and data was obtained from University College London Research Ethics Committee (REC reference 19181/001).

### Clinical study participants

We conducted a prospective observational study of patients aged ≥18 years who were undergoing major elective surgery at University College London (UCL) Hospitals between 1 August 2021 and 31 July 2022 requiring planned admission to the post-anesthetic care unit (10). Patient demographics, clinical data (physiology and diagnoses), laboratory data, and clinical outcomes were recorded from electronic healthcare records. We calculated the post-operative risk of death using the surgical outcome risk tool (SORT) score (11). The presence of a post-operative infection was adjudged by the treating clinical team based on clinical and laboratory data. We retrospectively assessed if patients met objective criteria of post-operative infections as defined by the standardized endpoints in perioperative medicine–core outcome measures for perioperative and anesthetic care (StEP-COMPAC) criteria (12). Patients were followed up to hospital discharge/death.

We conducted an exploratory sub-study of patients undergoing major surgery as part of a larger study investigating biomarkers in sepsis (REC reference 20/LO/1024). Based on healthy volunteers demonstrating a mean monocyte HLA-DR median fluorescence intensity (MFI) of 10,000 ± 2,500, with a power of 80%, and alpha of 0.05, a sample size of 25 would be required to detect a statistically significant difference of 20% between the two groups. We estimated sample size based on monocyte HLA-DR being reproducibly associated with mortality among critically ill patients with sepsis (8).

#### Sample processing

Following patient recruitment, venesection was performed at induction of anesthesia and 24 h after surgery and 8 mL of blood drawn into a cell preparation tube with sodium heparin (CPT™) vacutainer and a further 5 mL drawn into a serum tube [Beckton Dickinson (BD), Wokingham, UK]. Samples were processed within 1 h of venesection. CPT™ vacutainers were centrifuged at 1,500 g for 15 min at room temperature and the peripheral blood mononuclear cell (PBMC) layer extracted, washed twice in 2 mL of phosphate-buffered saline (PBS) before being resuspended in freezing media (fetal bovine serum [FBS; Gibco, Thermo Fisher (TF), Cambridge, UK] with 10% dimethyl sulfoxide (Sigma) and frozen to −80°C in a Mr Frosty™ and transferred to liquid nitrogen within 48 h for long-term storage.

Samples were analyzed in batches. Frozen PBMCs were defrosted by resuspension in RPMI Glutamax medium (Gibco) with 10% FBS (TF), washed once in media, counted (Countess 3 Automated cell counter, TF), and diluted to a concentration of 1 × 10^6^/mL. PBMCs were either labeled with antibodies for flow cytometry or cultured for *in vitro* stimulation.

### *In vitro* stimulation

For *in vitro* stimulation, PBMCs (1 × 10^6^/mL) were plated into 96-well plates (VWR, Lutterworth, UK) and incubated at 37°C, 5% CO_2_ with either heat-killed *E. coli* 0111:B4 (HKB, TF) at a concentration of 1 × 10^8^/mL for 12 h (for monocyte analysis) or CD3-28 beads (Miltenyi Biotec, Woking, UK) at a concentration of 4:1 for 48 h (for lymphocyte analysis). Following incubation, plates were centrifuged at 400 g for 5 min at room temperature in preparation for cell staining.

### Flow cytometry

To assess monocyte cell surface antigens, PBMCs were resuspended in PBS and incubated with relevant antibodies [CD14, CD16, HLA-DR, CD-80, CD-86, and CD274 (PD-L1)] and viability stain (Aqua UV Live/Dead). To assess lymphocyte viability and cell surface antigens, PBMCs were resuspended in annexin buffer (BD) and relevant antibodies [CD3, CD4, CD8, CD19, CD25 (IL-2RA), CD28, CD127 (IL-7RA), CD152 (CTLA-4), CD274 (PD-L1), and CD279 (PD-1)] with viability stain (Aqua UV Live/Dead and Annexin V). Details of products and concentrations used are detailed in **Supplementary Table 1**.

Intracellular cytokines were assessed by resuspending PBMCs in PBS and incubated with relevant cell surface antibodies and viability stain (Blue UV Live/Dead). After 30 min, PBMCs were fixed and permeabilized using the CytoFix/Perm kit (BD) after which they were resuspended and incubated in Cytoperm/Wash (BD) with antibodies to intracellular cytokines. Cells were acquired on an LSR II or Fortessa X20 flow cytometer (BD) running BD FACSDiva version 9.

Calibrations beads (BD) were run prior to each experiment, and compensation controls were applied to all samples prior to analysis. Single-stained unstimulated healthy donor cells were used as compensation controls for cell surface markers. Healthy donor cells were heat-treated at 60°C for 10 min as a positive control for cell death. Compensation beads (BD) were used as positive controls for intracellular cytokines. Fluorescence-minus-one samples for all fluorophores were used to identify cell populations. Cell populations of interest were identified using the following Boolean gating strategy: lymphocytes or PBMCs, singlets, live cells, and cell surface markers and stopping gate set at 10,000 events for either CD14^+^ monocytes or CD4^+^ lymphocytes.

Flow cytometry data were analyzed using FlowJo (version 10.7.1, BD). Samples with cell counts fewer than 50 in the population of interest were excluded.

### ELISA

We measured levels of IL-1β, IL-6 IL-10, TNF-α, PD-1, and PD-L1 in patient serum using Duoset ELISA kits (R&D Systems, Minneapolis, MN) as per the manufacturer’s instructions. Samples were diluted 1:2 in reagent dilutant. Optical densities were acquired on a SPECTROstar Nano microplate reader (BMG Labtech, Aylesbury, UK).

### Statistics

Clinical and demographic data are presented either as median (inter-quartile range) or number (percentage). Flow cytometry data are presented as either MFI (arbitrary units) or percentage positive cells with interquartile ranges. Continuous data were analyzed using Kruskal–Wallis test or Mann–Whitney U-test for comparison of more than two or two groups, respectively, whereas chi-squared test was used for analysis of categorical data.

Mixed-effects two-way ANOVA was used to assess the difference in continuous data over time (before and 24 h after surgery) between patients with and without subsequent infections. Data are presented as differences over time, between groups, and the difference in the change over time between the two groups (interaction term).

To assess if patients undergoing surgery demonstrate an immune signature, we undertook a principal component analysis (PCA) of 62 immune markers, age, and body mass index (BMI) for all patients for whom full datasets were available. Immune markers consisted of nine serological markers, nine monocyte markers, six CD4 lymphocyte markers, and six CD8 lymphocyte markers. Each immunological marker was assessed prior to and 24 h following surgery.

To identify statistically significant discriminators between patients with and without subsequent infections, we conducted multiple comparisons using a Mann–Whitney test and calculated a corrected p-value (−log10) with a false discovery rate (FDR) of 1% using a two-stage step-up method of Benjamini, Krieger, and Yekutieli, and area under the receiver operating characteristic curve (AUROC); and data are presented using a volcano plot. Graphs were constructed, and statistical analysis was performed using Prism (version 10, GraphPad, San Diego, CA). We conducted a regression analysis to assess independent risk factors associated with post-operative infection, using SPSS version 29.0 (IBM, Armonk, NY).

## Results

### Study participants

**Figure 2:**
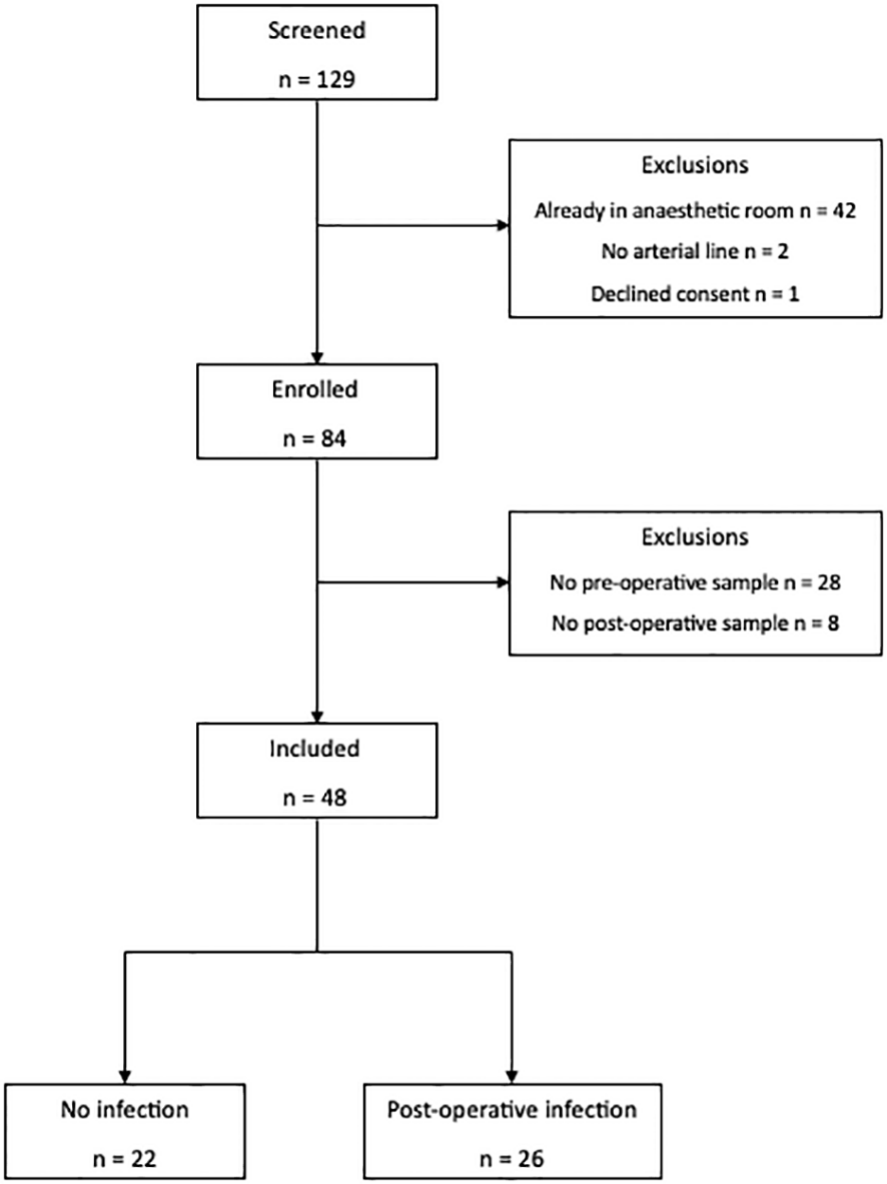
Flow diagram of patients screened and included in the study.

Forty-eight patients and 16 healthy volunteers were recruited (**Figure 2**). Details of missing data are provided in **Supplementary Table 2**. There was no difference in age (68 vs. 66), sex (64% vs. 79% male), BMI (24.73 vs. 25.23), American Society of Anesthesiologists (ASA) grade (2 vs. 3), co-morbidities, surgical site, use of dexamethasone for postoperative nausea and vomiting prophylaxis, or use of or duration of peri-operative antibiotics between patients who did or did not develop a post-operative infection. All patients received their first dose of prophylaxis within 30 min prior to skin incision (**Table 1**). Compared to healthy volunteers, patients were older (67 vs. 36; p < 0.0001), although sex (72% vs. 66%) and BMI were similar (25.0 vs. 23.54).

**Table 1:** Baseline demographics.

Variable	Healthy volunteer (n = 16)	No post-op infection (n = 22)	Post-op infection (n = 26)	p-value (patient groups only)
**Age (years)**	36 (35–38)	68 (56–72)	66 (56–72)	0.9556
**Gender (% male)**	66%	64%	79%	0.7749
**BMI**	23.54	24.73	25.23	0.5426
Co-morbidities				
Hypertension (%)	–	41%	27%	0.3057
Cardiovascular disease (%)	-	23%	19%	0.7663
Respiratory disease (%)	–	27%	38%	0.4126
Type 2 diabetes (%)	-	9%	19%	0.3213
ASA Grade (%)	–	2 (2–3)	3 (2–3)	0.3561
Active cancer (%)	-	64%	96%	0.0822
Cancer staging	–	1 (2–3)	2 (2–3)	0.0261
Neoadjuvant chemotherapy (%)	-	36%	67%	0.0822
Other immunosuppressive medication (%)	–	9%	8%	0.8613
Long-term steroids	-	9%	4%	0.4545
**SORT score (%)**	–	0.47 (0.25–0.87)	1.48 (0.37–3.17)	0.0169
Type of surgery				
Upper GI (%)	–	50%	75%	0.3218
Lower GU (%)	-	27%	21%	
Maxillofacial (%)	–	9%	13%	
Gynecological (%)	-	9%	0%	
Other (%)	–	5%	0%	
Peri-operative antibiotics				
Prophylaxis administered (%)	–	95%	100%	0.2931
Duration of prophylaxis (days)	-	1 (0–1)	1 (0–1)	0.7640
**Intra-operative dexamethasone use (%)**	–	95%	84%	0.2206
**Operation duration (min)**	-	174 (112–280)	287 (204–350)	0.0138
**Blood loss (mL)**	–	500 (500–500)	500 (100–500)	0.2019
**Peri-operative blood transfusion (%)**	-	5%	4%	0.9038
**Unplanned ICU readmission (%)**	–	5%	19%	0.1253
**Clavien–Dindo classification**	-	1 (0–1)	2 (2–3a)	<0.0001
**Hospital length of stay (days)**	–	8 (7–13)	15 (11–27)	0.0001
**Death (%)**	-	9%	4%	0.4545

Continuous data were analyzed using Mann–Whitney U-test for comparison of two groups, whereas chi-squared test was used for analysis of categorical data. BMI, body mass index; ASA, American Society of Anesthesiologists; GI, gastrointestinal; GU, genitourinary; ICU, intensive care unit. Blood loss was categorized as <100, 100–500, 500–1,000, 1,000–2,000, or >2,000 mL. For analysis, the higher value of the range was used.

Twenty-six (54%) patients developed a post-operative infection as defined by the StEP-COMPAC criteria (12). Site of infection included pneumonia (n = 17), wound (n = 4), anastomotic leak (n = 2), urinary tract (n = 2), and unknown (n = 1; this patient had clinical features consistent with an infection including fever; however, the source of the infection was unknown). Infections were diagnosed a median of 3 (2–4) days following surgery, with 10 patients having positive microbial cultures (**Supplementary Table 3**). Patients who developed an infection were more likely to have cancer (p = 0.0822), with distant spread (p = 0.0261), and receiving neoadjuvant chemotherapy (p = 0.0822). Their perioperative risk of mortality as measured by SORT score was higher (p = 0.0169) and operative times were longer (p = 0.0138). Patients who developed an infection had a longer hospital length of stay (p = 0.0001) although there were no differences in unplanned ICU readmission or mortality (**Table 1**). Antibiotic duration was a median of 5 (3–12) days for treatment of post-operative infections.

### Changes to immune cell phenotype 24 h following major surgery

**Figure 3:**
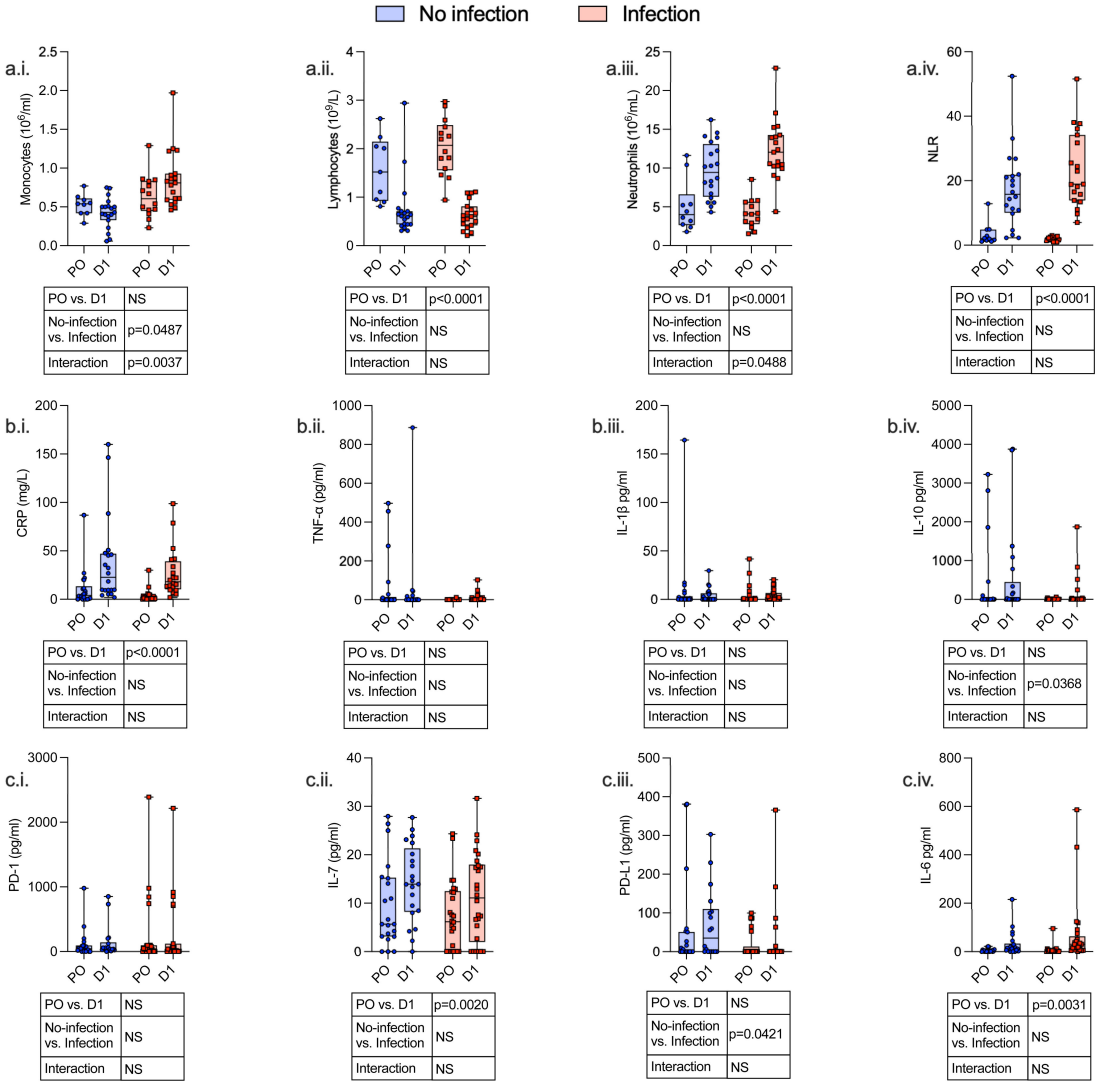
Serum and hematological variables identify an immunosuppressive phenotype both pre- and post-operatively, which is associated with the development of subsequent perioperative infections. Whole blood and serum were taken from patients undergoing major surgery at induction of anesthesia [pre-op (PO)] and 24 h post-operatively (D1) and analyzed by clinical laboratory evaluation whole-blood monocyte count (a.i.), lymphocyte count (a.ii.), neutrophil count (a.iii.) and neutrophil/lymphocyte ratio (NLR) (a.iv.), and CRP (b.i.). The following serum levels were measured by ELISA: TNF-α (b.ii.), IL-1β (b.iii.), IL-10 (b.iv.), PD-1 (c.i.), IL-7 (c.ii.), PD-L1 (c.iii.), and IL-6 (c.iv.). Data are expressed as either count or concentration. Dots represent individual patients, horizontal bar represents the median, box represents the interquartile range, and whisker represents the range. Data are analyzed using mixed-effects two-way ANOVA. Data are presented as differences over time, between groups, and the difference in the change over time between the two groups (interaction term). Only p-values < 0.05 shown. ns, not significant.

**Figure 4:**
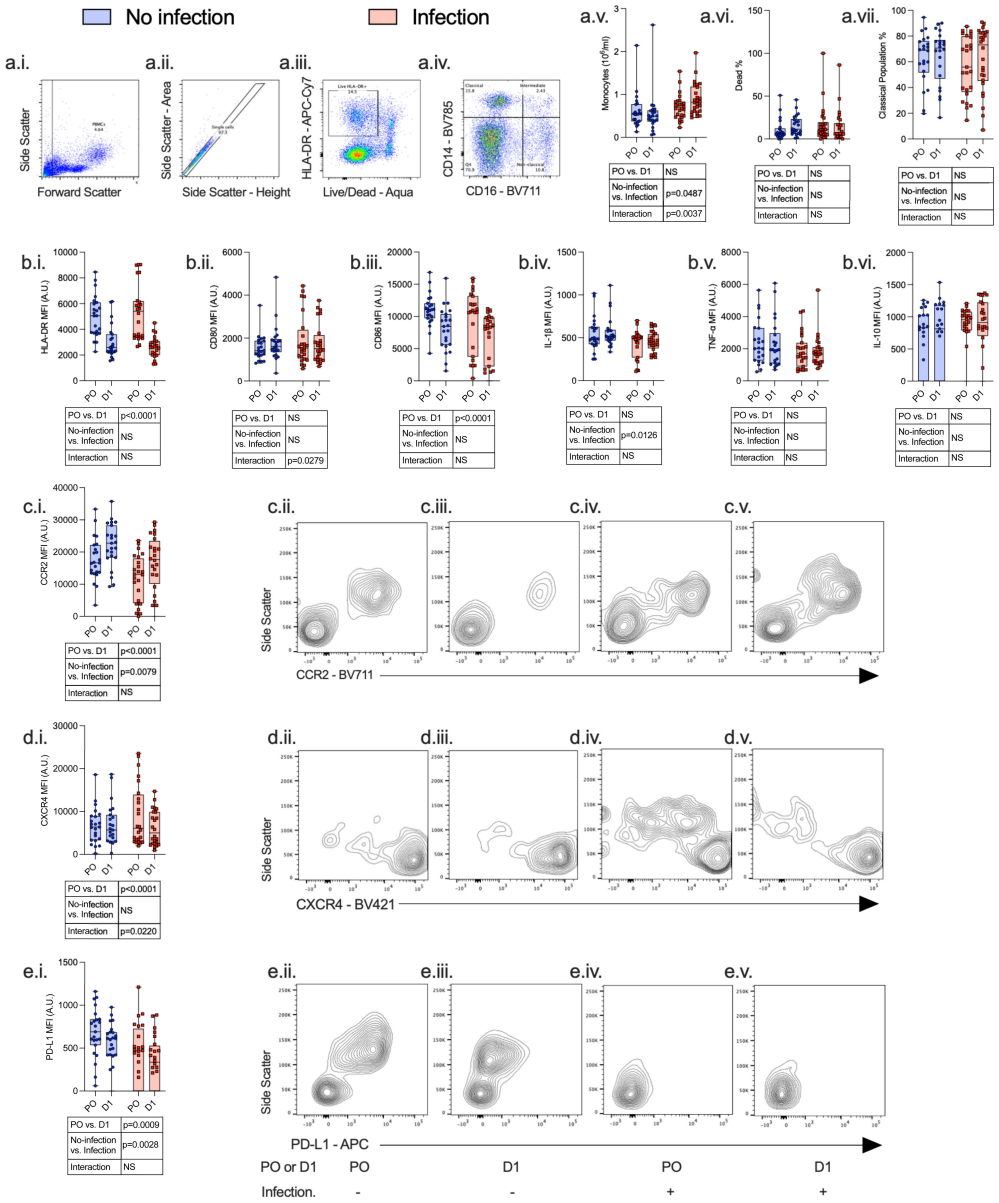
Monocytes express an immunosuppressive phenotype both pre- and post-operatively, which is associated with the development of subsequent perioperative infections. PBMCs were isolated from patients undergoing major surgery at induction of anesthesia [pre-op (PO)] and 24 h post-operatively (D1) and analyzed by flow cytometry with the following gating strategy: PBMCs, single cells, Live HLA-DR^+^ cells, and CD14/CD16 differentiation (a.i.–a.iv.). The following data were analyzed: whole-blood monocyte count (a.v), percentage dead (a.vi), percentage classical population (a.vii.), HLA-DR (b.i.), CD80 (b.ii.), CD86 (b.iii.), IL-1β (b.iv.), TNF-α (b.v.), IL-10 (b.vi.), CCR2 (c.i.–c.v.), CXCR4 (d.i.–d.v.), and PD-L1 (e.i.–e.v.). Data are expressed as either median fluorescence intensity (MFI) measured in arbitrary units (A.U.) or percentage of population (%). Dots represent individual patients, horizontal bar represents the median, box represents the interquartile range, and whisker represents the range. Data are analyzed using mixed-effects two-way ANOVA. Data are presented as differences over time, between groups, and the difference in the change over time between the two groups (interaction term). Only p-values < 0.05 shown. ns, not significant.

**Figure 5:**
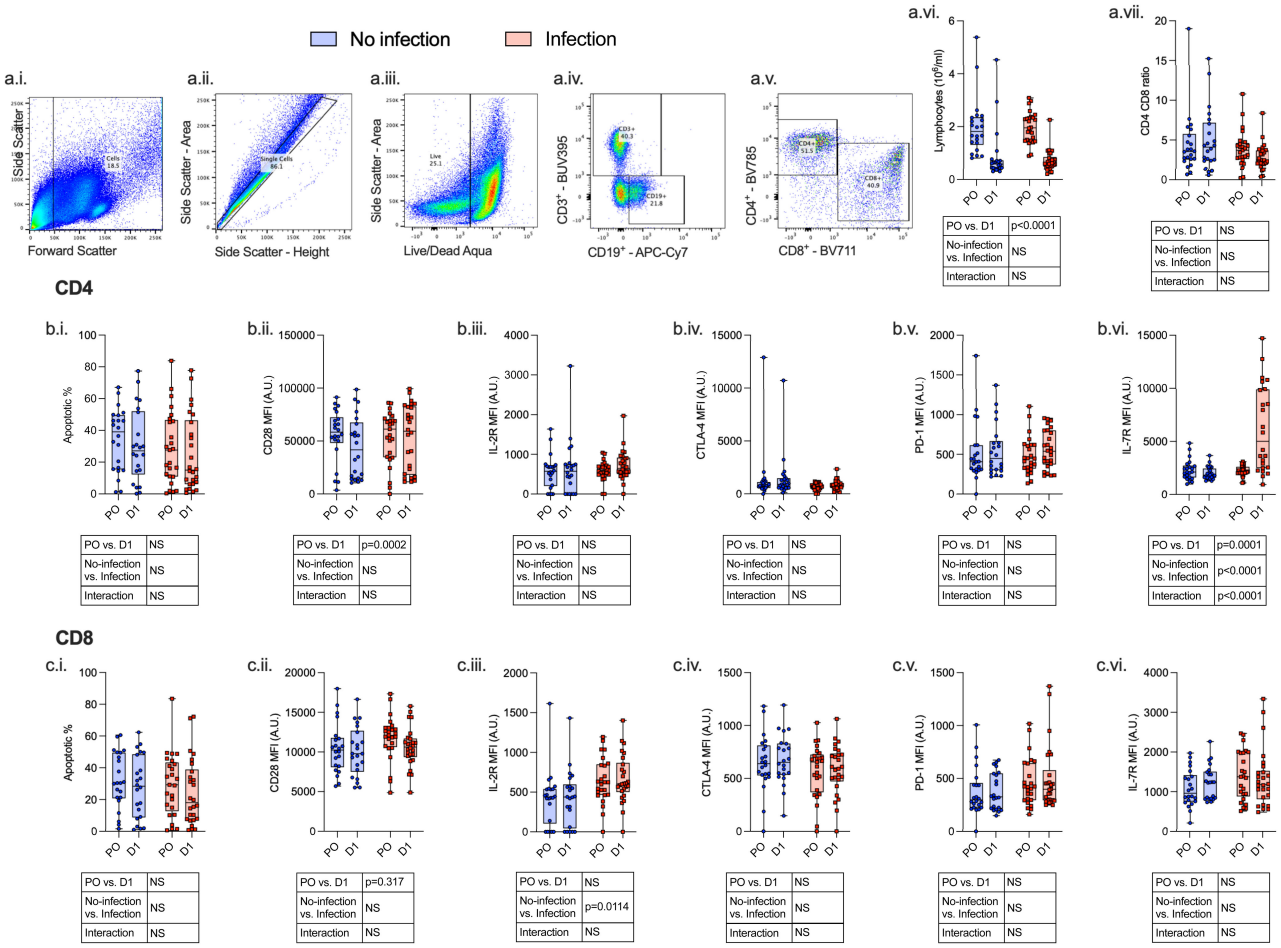
Lymphocytes express an immunosuppressive phenotype both pre- and post-operatively which is associated with the development of subsequent perioperative infections. PBMCs were isolated from patients undergoing major surgery at induction of anesthesia (pre-op; PO) and 24 h post-operatively (D1) and analyzed by flow cytometry with the following gating strategy: Lymphocytes, single cells, CD3^+^ or CD19^+^ cells, CD4^+^/CD8^+^ differentiation (a.i.–a.v.). The following data were analyzed: whole-blood lymphocyte count (a.vi), CD4^+^:CD8^+^ ratio (a.vii), CD4^+^ (row b.) and CD8^+^ (row c.) apoptosis (i.), CD28 (ii.), IL-2R (iii.), CTLA-4 (iv.), PD-1 (v.), and IL-7R (vi.). Data are expressed as either median fluorescence intensity (MFI) measured in arbitrary units (A.U.) or percentage of population (%). Dots represent individual patients, horizontal bar represents the median, box represents the interquartile range, and whisker represents the range. Data are analyzed using mixed-effects two-way ANOVA. Data are presented as differences over time, between groups, and the difference in the change over time between the two groups (interaction term). Only p-values < 0.05 shown. ns, not significant.

Twenty-four hours following surgery, there was a significant fall in lymphocyte count (p < 0.0001) and rise in neutrophil count (p < 0.0001), neutrophil:lymphocyte ratio (p < 0.0001), C-reactive protein (CRP) (p < 0.0001), serum IL-7 (p = 0.0020), and serum IL-6 (p = 0.0031) among patients with and without post-operative infections. There was a significant increase in monocyte CCR2 (p < 0.0001) expression and decreased CXCR4 (p < 0.0001), PD-L1 (p = 0.0009), HLA-DR (p < 0.0001), and CD86 (p < 0.0001) expression. CD4^+^ lymphocytes demonstrated a decrease in CD28 expression (p = 0.0002) and an increase in IL-7R (p = 0.0001) expression, whereas CD8^+^ lymphocyte expression of CD28 was decreased (p = 0.0317) (**Figures 3**–**5**; **Supplementary Table 4**).

Compared to patients who did not have a post-operative infection, patients who developed an infection had a higher monocyte count (p = 0.0487), higher CD4^+^ lymphocyte IL-7R (p < 0.0001) and CD8^+^ lymphocyte IL-2R (p = 0.0114) expression, and lower monocyte CCR2 (p = 0.0079) and PD-L1 (p = 0.0028) expression before and after surgery.

We next assessed surgery-induced immune perturbations across 24 h in patients who did and did not develop a post-operative infection. Compared to patients who did not have any post-operative infection, patients who developed an infection had an increase in monocyte count (p = 0.0037) and CD4^+^ lymphocyte IL-7R (p < 0.0001) and a decrease in monocyte CD80 (p = 0.0279) and CXCR4 expression (p = 0.0220) (**Figures 4**, **5**; **Supplementary Table 4**).

**Figure 6:**
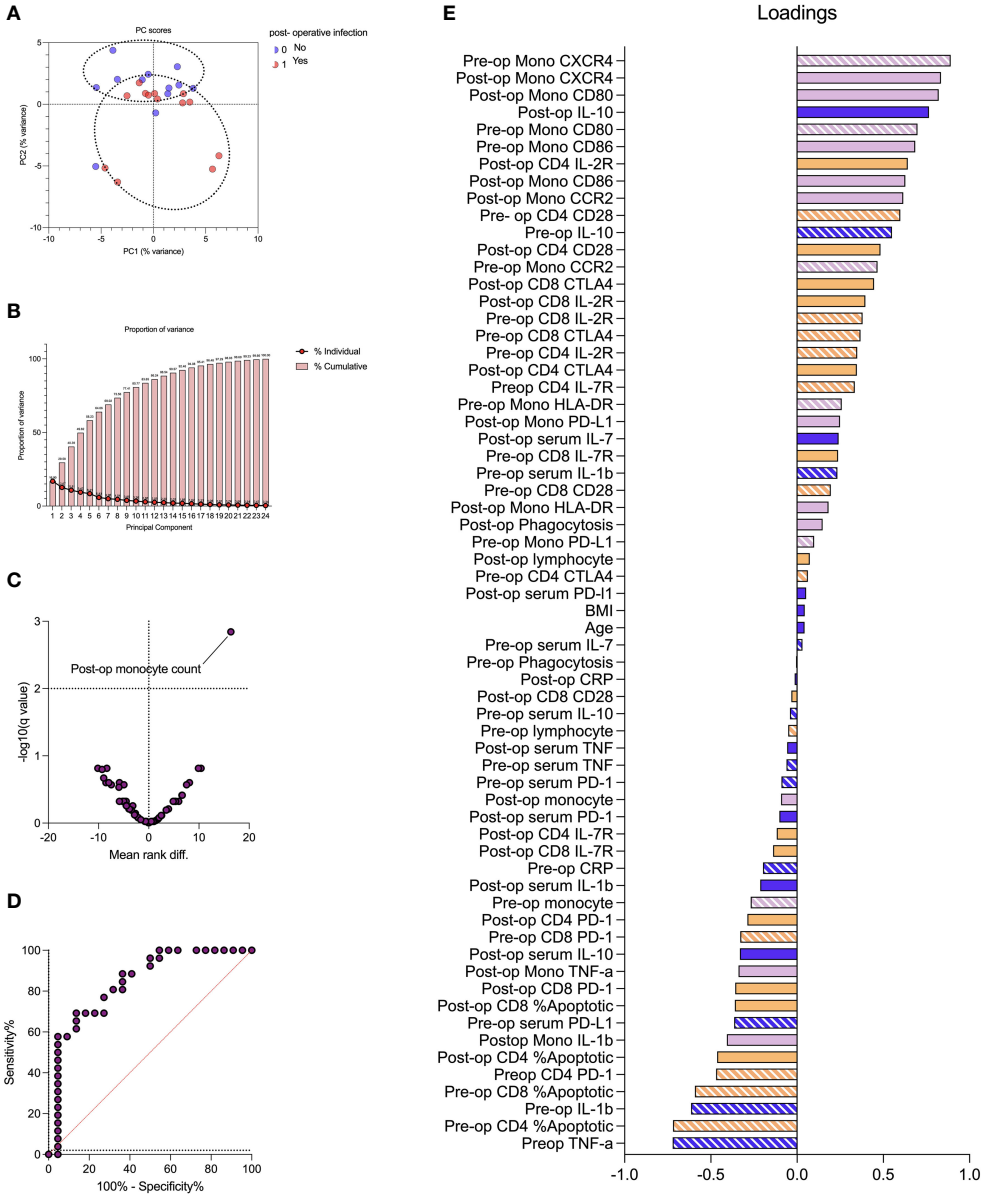
Impaired monocyte chemotaxis and antigen presentation are associated with the development of perioperative infection. Principal component analysis (PCA) demonstrated reasonable separation between patients with and without subsequent infections **(A)** with the first two components providing 30% cumulative proportion of variance **(B)**. The volcano plot demonstrated that post-operative monocyte count correlated with the development of post-operative infection **(C)** with an area under the receiving operator characteristic curve (AUROC) of 0.84 (p < 0.0001). The PCA loading plot **(E)** identified markers of monocyte chemotaxis (pre- and post-operative monocyte CXCR4) and antigen presentation (CD80) were greatest discriminators between patients with and without post-operative infections (loading vector coefficient of > 0.8). Pre-operative variables represented by stripes, and post-operative variables represented by solid fill. Dark blue represents clinical, serum, and hematological variables; purple represents monocyte variables; and orange represents T-lymphocyte variables. Data are analyzed by multiple Mann–Whitney tests (volcano plot) or principal component analysis (remaining plots).

PCA was conducted in 25 of 48 patients for whom full datasets were available. PCA provided reasonable separation between patients with and without subsequent infections with the first two components providing 30% cumulative proportion of variance. Monocyte chemokine receptors (pre- and post-operative monocyte CXCR4) and receptors involved in antigen presentation (CD80) were the greatest discriminators between patients with and without post-operative infections (loading vector coefficient of > 0.8). Post-operative monocyte count was significantly associated with the development of post-operative infection (FDR < 1%; adjusted p-value = 0.001) with an AUROC of 0.84 (p < 0.0001) (**Figure 6**).

Covariates included in the multivariate analysis to assess for risk factors associated with post-operative infection were based on univariate analyses. We included post-operative monocyte count, age, the presence of active cancer, and surgical time in the regression analysis. Active cancer (OR = 24.6; p = 0.056) and post-operative monocyte count (OR = 8.9, p = 0.056) were associated with increased risk of post-operative infections, albeit not statistically significant. Age (OR = 1.019; p = 0.593) and surgical time (OR = 1.004; p = 0.327), however, were not independently associated with post-operative infections (**Supplementary Table 5**).

### *In vitro* functional capacity of PBMCs before and after surgery

Analysis of immune cell phenotype before and after surgery provided insight into changes associated with surgery. Next, we sought to investigate the effect of surgery on the ability of immune cells to respond to an *in vitro* stimulus (i.e., their functional capacity). PBMCs isolated from healthy volunteers were used as a reference.

### Monocyte stimulation

**Figure 7:**
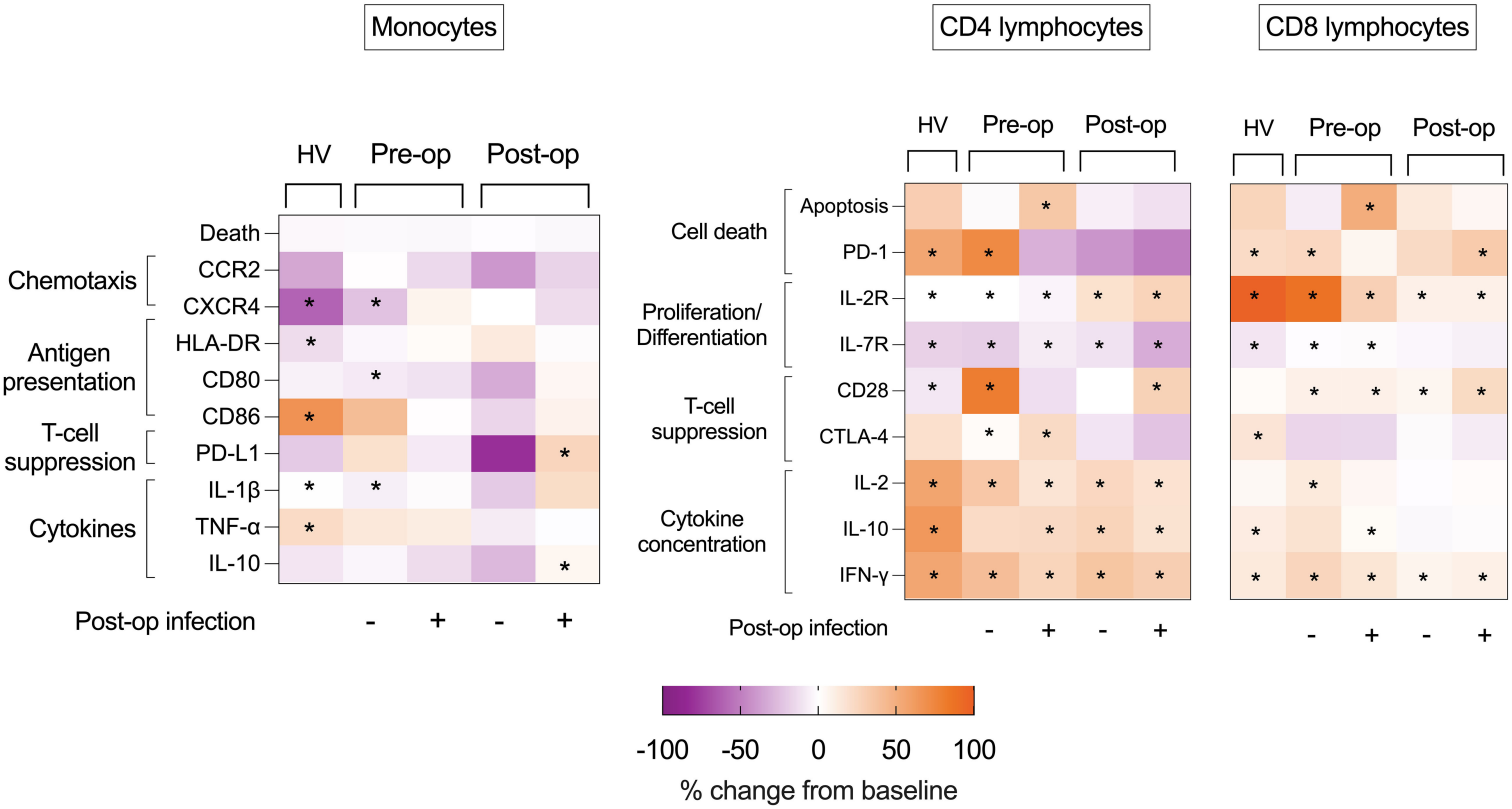
Heat maps of effect of ex vivo stimulus on volunteer and patient immune cells. Heat maps illustrating percentage changes in immune cell phenotype following HKB (monocyte) or CD3-CD28 bead (lymphocyte) stimulation in PBMCs isolated from healthy volunteers (HV) or from pre- and post- operative samples obtained from patients who did or did not succumb to post- operative infectious complications. Difference between HKB-stimulated compared to unstimulated cells analyzed using Mann–Whitney U-test, and displayed as percentage change, only p < 0.05 shown indicated by *.

Following 24 h of stimulation with HKB, there was an increase in CD86 (p = 0.052), IL-1β (p = 0.005), and tumour necrosis factor alpha (TNF-α) (p = 0.0049) and a reduction in CXCR4 (p = 0.0005) and HLA-DR (p = 0.001) in healthy volunteer monocytes. Among patients without a post-operative infection, there was an increase in monocyte IL-1β (p = 0.0141) and a reduction in CXCR4 (p = 0.004) in pre-operative samples. Following surgery, there were no changes in monocyte phenotype following HKB stimulus. Among patients with a post-operative infection, there were no changes in monocyte phenotype following HKB stimulus in pre-operative samples. Following surgery, HKB induced an increase in monocyte PD-L1 expression (p = 0.003) and reduction in IL-10 (p = 0.0255) (**Figure 7**; **Supplementary Table 4**, **Supplementary Figure 1**).

### CD4^+^ lymphocyte stimulation

Following PBMC stimulation with CD3-CD28 beads, an increase in CD4^+^ lymphocyte IL-2R expression and a reduction in IL-7R expression were consistent between healthy volunteers, pre-operative and post-operative samples (in patients with and without post- operative infections) (p < 0.05 for all). An increase in CD4^+^ lymphocyte CD28 expression was seen in all groups (p < 0.05 for all) apart from pre-operative samples from patients with post-operative infections. CTLA-4 expression increased only in pre-operative PBMCs isolated from patients with (p = 0.0322) and without (p = 0.0283) subsequent infections.

An increase in CD4^+^ lymphocyte PD-1 was limited to healthy volunteers (p = 0.0024) and in pre-operative PBMCs from patients without post-operative infection (p = 0.0032), although an increase in CD4^+^ lymphocyte apoptosis was not observed. In contrast, there was in increase in CD4^+^ lymphocyte % apoptosis (p = 0.0059) among pre-operative samples from patients with post-operative infections, but no increase in PD-1 expression (**Figure 7**; **Supplementary Figure 2**).

### CD8^+^ lymphocyte stimulation

Following PBMC stimulation with CD3-CD28 beads, an increase in CD8^+^ lymphocyte IL-2R expression was consistent between healthy volunteers, pre-operative and post-operative samples (in patients with and without post-operative infections) (p < 0.05 for all). An increase in CD8^+^ lymphocyte CD28 expression was seen in patients before and after surgery (with and without post-operative infections) (p < 0.05 for all), but not in healthy volunteers. In contrast, an increase in CTLA-4 was seen in healthy volunteers (p = 0.0210) but not in PBMCs isolated from patients before and after surgery. A reduction in IL-7R expression was evident only in healthy volunteers (p = 0.0005) and in pre-operative PBMCs isolated from patients with (p = 0.0277) and without (p = 0.0275) subsequent infections. An increase in CD8^+^ lymphocyte PD-1 expression was seen in healthy volunteers (p = 0.001), in pre-operative CD8^+^ lymphocytes from patients without infectious complications (p = 0.0082) and post-operative CD8^+^ lymphocytes from patients with infectious complications (p = 0.0060) (**Figure 7**; **Supplementary Table 4**; **Supplementary Figure 2**).

## Discussion

We demonstrate reduced baseline pre- and post-operative monocyte CXCR4 and CD80 expression (chemokine receptors and co-stimulation markers, respectively) in patients who subsequently developed an infection as well as a profound and selective post-operative increase in CD4^+^ lymphocyte IL-7 receptor expression in the infection group only. Higher post-operative monocyte count was significantly associated with the development of post-operative infection (FDR < 1%; adjusted p-value = 0.001) with an area under the receiver operating characteristic curve of 0.84 (p < 0.0001).

Major surgery represents a significant and prolonged physiological and inflammatory insult. Our understanding of how the immune response becomes perturbed in this context remains limited, although several reports how shown that innate and adaptive cells can mount opposing pro-inflammatory functions and anti-inflammatory functions (13–15). We therefore hypothesize that patients in whom immunosuppression is profound are those most likely to develop infection. We found that changes in immune phenotype within 24 h of major surgery were associated with the development of a subsequent infection.

A significant reduction in receptors involved in monocyte antigen presentation (CD80) and chemokine receptors (CXCR4) was associated with infectious complications. Monocyte CCR2 expression increased following surgery, in patients with and without post-operative infections. However, patients who developed post-operative infections had lower levels of CCR2 expression pre- and post-operatively compared to patients without post-operative infections. Circulating monocyte counts fell in patients who did not develop any post-operative infections, whereas this was not evident in patients who developed a post-operative infection. Together, these findings suggest an impairment in monocyte chemotaxis to sites of inflammation or infection are associated with post-operative infections. Post-operative complications in high risk and cancer surgery are associated with higher peripheral monocyte counts (16, 17). The mechanism underpinning this observation is unclear, but may represent an impairment in ability of monocytes to migrate to sites of inflammation/infection akin to impairments in neutrophil chemotaxis associated with post-operative infections (18–21).

Lymphopenia and impaired lymphocyte function are associated with increased risk of developing post-operative infections (22). We found a significant reduction in lymphocyte count and expression of lymphocyte co-stimulatory receptor (CD28) following surgery, although this did not discriminate between patients with and without post-operative infections. Alternative pathways associated with lymphocyte dysfunction may be contributary (22).

Several studies have investigated the change in immunophenotype of patients following surgery (4, 5, 13–15, 23, 24). However, few studies have investigated the effect on the dynamic immune response to a subsequent *in vitro* challenge before and after surgery and how this differs between patients who develop post-operative infections and those who do not.

In comparison to healthy volunteer monocytes, there was paucity of changes in immune phenotype following *in vitro* HKB stimulus in patients undergoing surgery. Although patients who did not develop post-operative infections demonstrated few changes consistent with healthy volunteers in the pre-operative period, monocytes failed to demonstrate any response to *in vitro* HKB stimulus following surgery, suggestive of decreased monocyte functional reserve seen after other major surgery (25–27).

Among patients who had a subsequent post-operative infection, there were no changes to monocyte phenotype following HKB stimulus in cells isolated pre-operatively. In contrast, post-operative monocyte PD-L1 expression increased following HKB stimulus in patients with post-operative infections, which was not evident in healthy volunteers or patients without post-operative infections. An increase in monocyte PD-L1 expression is associated with CD4^+^ lymphocyte inhibition via the PD-1/PD-L1 pathway suggesting a plausible mechanism (28). Elevated monocyte PD-L1 expression in critically ill patients is associated with lymphocyte anergy and increased risk of secondary infections (28, 29). Monocyte PD-L1 is less well characterized in surgery although elevated monocyte and serum PD-L1 are associated with increased risk of infection in other inflammatory processes such as pancreatitis (30, 31).

In contrast to monocytes, many changes to CD4^+^ and CD8^+^ lymphocyte immune phenotype following CD3-CD28 bead stimulation in healthy volunteer cells were seen in patient cells. This was more evident in pre-operative lymphocytes from patients without post-operative infections. Fewer changes were seen in post-operative samples compared to pre-operative samples, suggestive of reduced functional capacity following surgery. Impaired lymphocyte functional responses may be mediated by a hypometabolic phenotype which occurs following surgery (22).

A number of approaches have been attempted to reverse post-operative immunosuppression including granulocyte colony stimulating factor (G-CSF) (32), interferon gamma (IFN-γ) (33), IL-10 pathways (34) but with no conclusive benefit. Attempted modulation of a single immunomodulatory target is unlikely to yield results as related co-stimulatory or inhibitory pathways may be simultaneously affected.

We acknowledge limitations in our study. Assessment of the trajectory of immune phenotype over a longer duration would provide greater insight into the recovery following surgery. Majority of our patients had underlying solid organ malignancies, a proportion of who received chemotherapy. It is not possible to extrapolate our findings to other cohorts of patients. All *in vitro* experiments were performed using a single concentration and strain of HKB or CD3-CD28 beads. The percentage of T-lymphocyte apoptosis was high compared to healthy populations; however, this was consistent with other high-risk surgical cohorts (35). We have not investigated neutrophil function, which is also known to be impaired in surgery (18–21). Similarly, we have not investigated the role of B cells. We have not presented data on intermediate and non-classical monocyte subsets as cell counts from patients were limited. However, quantification of cell surface markers on monocyte subsets is rarely, if ever, used to stratify immune status in critically ill patients.

We assessed levels of ligands and receptors (e.g., PD-L1 and PD-1) on flow cytometry but were unable to assess their interaction or associated pathways. Specifically, monocyte chemokine receptor expression could be further explored using chemotaxis assays. However, typical cell counts required for such assays exceed that obtained from patients. The response to an *in vitro* stimulus (HKB of CD3-CD28 beads) may not represent *in vitro* changes in patients with infections.

We found that post-operative monocyte count is, by far, the most differentiating feature on the volcano plot although not a major discriminator on PCA. This might be explained by the fact that PCA includes only patients with complete datasets. We included 62 immune markers, age, and BMI; full datasets were available in 25 of 47 patients. However, for multiple comparisons using a Mann–Whitney test, data from all patients were used.

We conducted a multivariate analysis to assess the independent effects of different covariates on infectious complications. Due to the relatively small sample size, we were limited in the number of covariates in our analysis. However, the multivariate analysis supports the findings of our other analyses, demonstrating that post-operative monocyte count may be independently associated with post-operative infections.

An unsupervised analysis of a wider panel of markers may reveal other druggable targets. Several studies have assessed the transcriptomic profile of immune cells in the perioperative period, although transcriptional changes may not be reflected in cell surface proteins/receptor expression, and bulk transcriptomics do not directly assess the phenotype of specific cell subsets (4, 24).

A major strength of our study is the assessment of dynamic immune function, in addition to basal immune phenotype (36). We have demonstrated important differences in host response to surgery between patients who do and do not develop a subsequent infection. Given the numbers of patients who undergo major surgery globally and the proportion who develop post-operative infections, our findings warrant further investigations. Specifically, the underlying mechanisms and potential therapeutics to reverse defects in immune cell function require exploration.

## Data availability statement

The raw data supporting the conclusions of this article will be made available by the authors, without undue reservation.

## Ethics statement

The studies involving humans were approved by London – Queen Square Research Ethics Committee and University College London Research Ethics Committee. The studies were conducted in accordance with the local legislation and institutional requirements. The participants provided their written informed consent to participate in this study.

## Author contributions

TS: Writing – original draft, Writing – review & editing. AW: Writing – review & editing. RL: Writing – review & editing. FR: Writing – review & editing. AC: Writing – review & editing. NS: Writing – review & editing. RR: Writing – review & editing. JW: Writing – review & editing. AA-H: Writing – review & editing. AD: Writing – review & editing. MS: Writing – review & editing. DB: Writing – review & editing. NA: Writing – original draft, Writing – review & editing.

## University College London Hospitals Critical Care Research Team

Deborah Smyth, Georgia Bercades, Ingrid Hass, Alexandra Zapata Martinez, Laura Gallagher, and Gladys Martir.
