# Supplementary material for: Early dynamic changes to monocytes following major surgery are associated with subsequent infections
Source: Front Immunol. 2024 Apr 9;15:1352556. doi: 10.3389/fimmu.2024.1352556 (PMC11035723; doi:10.3389/fimmu.2024.1352556)
Supplement: Supplementary file 1 [file DataSheet_1.docx]

# Supplemental Material

| Supplemental Table 1: Flow cytometry fluorochromes used | | | |
| --- | --- | --- | --- |
| **Antibody** | **Fluorochrome** | **Catalogue no** | **[Final]** |
| CD14 | BV785 | BL 301840 | 1:250 |
| CD16 | BUV395 | BD 563785 | 1:250 |
| HLA-DR | APC-Cy7 | BL307618 | 1:250 |
| LD Aqua | Aqua UV | TF L34957 | 1:1000 |
| CD192 (CCR2) | BV711 | BL 357232 | 1:250 |
| CD184 (CXCR4) | BV421 | BL 306518 | 1:250 |
| CD80 | PE | BL 305208 | 1:250 |
| CD86 | PE-Dazzle | BL 374218 | 1:250 |
| CD274 (PD-L1) | APC | BD 563741 | 1:250 |
| IL-1β | FITC | TF 11-7018-42 | 1:100 |
| IL-10 | PE-CY7 | BL 501420 | 1:100 |
| TNF-α | BUV737 | TF 367-7349-42 | 1:100 |
| CD3 | BUV395 | BD 564001 | 1:250 |
| CD4 | BV785 | BL 317442 | 1:250 |
| CD8 | BV711 | BD 563677 | 1:250 |
| CD19 | APC-Cy7 | BD 557791 | 1:250 |
| LD Blue | Blue UV | TF L34962 | 1:1000 |
| IL-2 | PE | BL 500307 | 1:100 |
| IFN-y | BV510 | BL 502930 | 1:100 |
| Annexin | AF 350 | TF A23202 | 1:250 |
| CD25 (IL-2R) | BV421 | BD 562442 | 1:250 |
| CD28 | BUV737 | BD 748475 | 1:250 |
| CD127 (IL-7R) | PE | BL 351304 | 1:250 |
| CD152 (CTLA-4) | PE-Dazzle | BL 349922 | 1:250 |
| CD279 (PD-1) | PE-Cy7 | BD 561272 | 1:250 |
| **Abbreviations:** CD: Cluster of Differentiation; HLA-DR: Human leukocyte antigen – DR isotype; LD: Live/Dead; CCR2: C-C motif chemokine receptor 2; CXCR4: CXC motif chemokine receptor 4; IL: Interleukin; TNF: Tissue necrosis factor; PD-L1: Programmed death ligand 1; IFN: Interferon; BL: Biolegend; BD: Beckton Dickinson; TF: Thermo Fisher | | | |

| **Supplemental Table 2: Missing data** | | | | | | | | | | | | | | | | | |
| --- | --- | --- | --- | --- | --- | --- | --- | --- | --- | --- | --- | --- | --- | --- | --- | --- | --- |
| Study ID | Demographics | Laboratory data | | ELISA | | Monocyte unstimulated | | Monocyte stimulated | | CD4 unstimulated | | CD4 stimulated | | CD8 unstimulated | | CD8 stimulated | |
|  |  | PO | D1 | PO | D1 | PO | D1 | PO | D1 | PO | D1 | PO | D1 | PO | D1 | PO | D1 |
| 030 |  |  |  |  |  |  |  |  |  |  |  |  |  |  |  |  |  |
| 035 |  |  |  |  |  |  |  |  |  |  |  |  |  |  |  |  |  |
| 040 |  |  |  |  |  |  |  |  |  |  |  |  |  |  |  |  |  |
| 042 |  |  |  |  |  |  |  |  |  |  |  |  |  |  |  |  |  |
| 043 |  |  |  |  |  |  |  |  |  |  |  |  |  |  |  |  |  |
| 048 |  |  |  |  |  |  |  |  |  |  |  |  |  |  |  |  |  |
| 053 |  |  |  |  |  |  |  |  |  |  |  |  |  |  |  |  |  |
| 054 |  |  |  |  |  |  |  |  |  |  |  |  |  |  |  |  |  |
| 057 |  |  |  |  |  |  |  |  |  |  |  |  |  |  |  |  |  |
| 059 |  |  |  |  |  |  |  |  |  |  |  |  |  |  |  |  |  |
| 061 |  |  |  |  |  |  |  |  |  |  |  |  |  |  |  |  |  |
| 062 |  |  |  |  |  |  |  |  |  |  |  |  |  |  |  |  |  |
| 063 |  |  |  |  |  |  |  |  |  |  |  |  |  |  |  |  |  |
| 064 |  |  |  |  |  |  |  |  |  |  |  |  |  |  |  |  |  |
| 072 |  |  |  |  |  |  |  |  |  |  |  |  |  |  |  |  |  |
| 074 |  |  |  |  |  |  |  |  |  |  |  |  |  |  |  |  |  |
| 075 |  |  |  |  |  |  |  |  |  |  |  |  |  |  |  |  |  |
| 076 |  |  |  |  |  |  |  |  |  |  |  |  |  |  |  |  |  |
| 078 |  |  |  |  |  |  |  |  |  |  |  |  |  |  |  |  |  |
| 079 |  |  |  |  |  |  |  |  |  |  |  |  |  |  |  |  |  |
| 081 |  |  |  |  |  |  |  |  |  |  |  |  |  |  |  |  |  |
| 082 |  |  |  |  |  |  |  |  |  |  |  |  |  |  |  |  |  |
| 029 |  |  |  |  |  |  |  |  |  |  |  |  |  |  |  |  |  |
| 036 |  |  |  |  |  |  |  |  |  |  |  |  |  |  |  |  |  |
| 037 |  |  |  |  |  |  |  |  |  |  |  |  |  |  |  |  |  |
| 038 |  |  |  |  |  |  |  |  |  |  |  |  |  |  |  |  |  |
| 039 |  |  |  |  |  |  |  |  |  |  |  |  |  |  |  |  |  |
| 041 |  |  |  |  |  |  |  |  |  |  |  |  |  |  |  |  |  |
| 044 |  |  |  |  |  |  |  |  |  |  |  |  |  |  |  |  |  |
| 046 |  |  |  |  |  |  |  |  |  |  |  |  |  |  |  |  |  |
| 049 |  |  |  |  |  |  |  |  |  |  |  |  |  |  |  |  |  |
| 050 |  |  |  |  |  |  |  |  |  |  |  |  |  |  |  |  |  |
| 051 |  |  |  |  |  |  |  |  |  |  |  |  |  |  |  |  |  |
| 052 |  |  |  |  |  |  |  |  |  |  |  |  |  |  |  |  |  |
| 055 |  |  |  |  |  |  |  |  |  |  |  |  |  |  |  |  |  |
| 056 |  |  |  |  |  |  |  |  |  |  |  |  |  |  |  |  |  |
| 058 |  |  |  |  |  |  |  |  |  |  |  |  |  |  |  |  |  |
| 065 |  |  |  |  |  |  |  |  |  |  |  |  |  |  |  |  |  |
| 066 |  |  |  |  |  |  |  |  |  |  |  |  |  |  |  |  |  |
| 067 |  |  |  |  |  |  |  |  |  |  |  |  |  |  |  |  |  |
| 068 |  |  |  |  |  |  |  |  |  |  |  |  |  |  |  |  |  |
| 069 |  |  |  |  |  |  |  |  |  |  |  |  |  |  |  |  |  |
| 071 |  |  |  |  |  |  |  |  |  |  |  |  |  |  |  |  |  |
| 073 |  |  |  |  |  |  |  |  |  |  |  |  |  |  |  |  |  |
| 077 |  |  |  |  |  |  |  |  |  |  |  |  |  |  |  |  |  |
| 080 |  |  |  |  |  |  |  |  |  |  |  |  |  |  |  |  |  |
| 083 |  |  |  |  |  |  |  |  |  |  |  |  |  |  |  |  |  |
| 084 |  |  |  |  |  |  |  |  |  |  |  |  |  |  |  |  |  |
| Green: All data present, Yellow: Some data missing, Red: No data available | | | | | | | | | | | | | | | | | |

| Supplemental Table 3: Details of infections | | | | | |
| --- | --- | --- | --- | --- | --- |
| **Study ID** | **Days from surgery** | **Source** | **Met positive culture criteria** | **Met fever criteria** | **Met other source criteria** |
| 029 | 4 | Urine | E. coli, Enterococcus faecalis, Enterococcus avium |  |  |
| 036 | 2 | Chest |  |  | Yes |
| 037 | 3 | Wound |  |  | Yes |
| 038 | 2 | Chest |  |  | Yes |
| 039 | 5 | Chest |  |  | Yes |
| 041 | 0 | Chest |  |  | Yes |
| 044 | 10 | Wound | S. aureus |  |  |
| 046 | 5 | Chest | Pseudomonas |  |  |
| 049 | 2 | Wound | Pseudomonas |  |  |
| 050 | 2 | Chest |  |  | Yes |
| 051 | 1 | Chest |  |  | Yes |
| 052 | 4 | Chest |  |  | Yes |
| 055 | 5 | Chest | E. coli, Candida, Proteus |  |  |
| 056 | 3 | Chest |  |  | Yes |
| 058 | 2 | Wound | Candida, Klebsiella, Lactobacillus paracasei |  |  |
| 065 | 2 | Chest | S. aureus, Candida, Enterococcus faecalis |  |  |
| 066 | 2 | Chest |  |  | Yes |
| 067 | 2 | Chest |  |  | Yes |
| 068 | 9 | Chest |  |  | Yes |
| 069 | 4 | Chest |  |  | Yes |
| 071 | 4 | Urine | Pseudomonas |  |  |
| 073 | 2 | Chest | Enterobacter cloacae, Candida |  |  |
| 077 | 4 | Chest |  |  | Yes |
| 080 | 2 | Unknown |  | Yes |  |
| 083 | 1 | Chest | Candida |  |  |
| 084 | 3 | Chest |  |  | Yes |
| Diagnosis based on standardized endpoints in perioperative medicine – core outcome measures for perioperative and anaesthetic care (StEP-COMPAC) criteria | | | | | |

| **Supplemental Table 4: Summary of significant results** | | | | | | | | | |
| --- | --- | --- | --- | --- | --- | --- | --- | --- | --- |
| **Cell Type** | **Cell subtype** | **Functional association** | **Marker** | **Unstimulated p-value** | **Stimulated volunteer p-value** | **Stimulated pre-operative** | | **Stimulated post-operative** | |
|  |  |  |  |  |  | **No-infection p-value** | **Infection p-value** | **No-infection p-value** | **Infection p-value** |
| Monocytes |  |  | Count | 0.0481 | - | - | - | - | - |
|  | Classical |  | %Population | ns | 0.0425 | ns | ns | 0.0583 | ns |
|  |  |  | %Dead | ns | ns | ns | ns | ns | ns |
|  |  | Chemokine receptor | CCR2 | 0.0079 | ns | ns | ns | ns | ns |
|  |  |  | CXCR4 | ns | 0.0005 | 0.0039 | ns | ns | ns |
|  |  | Antigen presentation | HLA-DR | ns | 0.0010 | ns | ns | ns | ns |
|  |  |  | CD80 | ns | ns | 0.0355 | ns | ns | ns |
|  |  |  | CD86 | ns | 0.0522 | ns | ns | ns | ns |
|  |  | T-cell suppression | PD-L1 | 0.0028 | ns | ns | ns | ns | 0.0032 |
|  |  | Cytokine concentration | IL-1b | 0.0126 | 0.0005 | 0.0141 | ns | ns | ns |
|  |  |  | IL-10 | ns | ns | ns | ns | ns | 0.0225 |
|  |  |  | TNF-a | ns | 0.0049 | ns | ns | ns | ns |
| Lymphocyte |  |  | Count | ns | ns | ns | ns | ns | ns |
|  |  |  | CD4:8 ratio | ns | ns | 0.0093 | ns | ns | 0.0327 |
|  | CD4^+^ | Cell activation | CD28 | ns | ns | 0.0009 | ns | ns | <0.0001 |
|  |  |  | CTLA-4 | ns | ns | 0.0322 | 0.0283 | ns | ns |
|  |  | Proliferation & Differentiation | IL-2 | ns | 0.0005 | 0.0098 | 0.0103 | 0.0046 | 0.0342 |
|  |  |  | IL-2R | ns | 0.0005 | <0.0001 | 0.0321 | 0.0082 | <0.0001 |
|  |  |  | IL-7R | <0.0001 | 0.0005 | 0.0275 | 0.0277 | 0.0004 | 0.0254 |
|  |  | Cell death | PD-1 | ns | 0.0024 | 0.0032 | ns | ns | ns |
|  |  |  | Apoptosis | ns | ns | ns | 0.0059 | ns | ns |
|  |  | Cytokine | IFN-y | - | 0.0019 | 0.0003 | 0.0046 | 0.0041 | 0.0040 |
|  |  |  | IL-10 | - | 0.0068 | ns | 0.0244 | 0.0026 | 0.0028 |
|  | CD8^+^ | Cell activation | CD28 | ns | ns | 0.0009 | 0.0064 | 0.0419 | 0.0103 |
|  |  |  | CTLA-4 | ns | 0.0210 | ns | ns | ns | ns |
|  |  | Proliferation & Differentiation | IL-2 | ns | ns | 0.0130 | ns | ns | ns |
|  |  |  | IL-2R | 0.0114 | 0.0005 | <0.0001 | 0.0003 | 0.0085 | <0.0001 |
|  |  |  | IL-7R | ns | 0.0005 | 0.0275 | 0.0277 | ns | ns |
|  |  | Cell death | PD-1 | ns | 0.0010 | 0.0082 | ns | ns | 0.0060 |
|  |  |  | Apoptosis | ns | ns | ns | 0.0020 | ns | ns |
|  |  | Cytokine concentration | IFN-y | ns | 0.0005 | 0.0103 | 0.0003 | <0.0001 | 0.0181 |
|  |  |  | IL-10 | ns | 0.0674 | ns | 0.0441 | ns | ns |
| **Abbreviations:** CD: Cluster of Differentiation; HLA-DR: Human leukocyte antigen – DR isotype; CCR2: C-C motif chemokine receptor 2; CXCR4: CXC motif chemokine receptor 4; IL: Interleukin; TNF: Tissue necrosis factor; PD-L1: Programmed death ligand 1; IFN: Interferon; ns: Not significant. | | | | | | | | | |

| **Supplemental Table 5: Multivariate analysis** | | | |
| --- | --- | --- | --- |
| **Co-variate** | **Odds ratio** | **Confidence interval** | **p-value** |
| Post-operative monocyte count | 8.9 | 0.95 - 84 | 0.056 |
| Active cancer | 24.6 | 0.93 - 652 | 0.056 |
| Operation duration | 1.004 | 0.99 - 1.01 | 0.327 |
| Age | 1.019 | 0.95 - 1.09 | 0.593 |
| Co-variates used in the binary logistic regression multivariate analysis were based on univariate analysis. | | | |

### Supplemental Figure 1: In response to a bacterial infection *in vitro*, patient monocytes display an immunosuppressive phenotype.

PBMCs were isolated from healthy volunteer (green) and patients who did not (blue) and did (red) develop a post-operative infection and the effect of stimulation with heat-killed bacteria (HKB) on monocyte HLA-DR (a.i.), CD80 (a.ii.), and CD86 (a.iii.) expression, IL-10 (b.i.), IL-1β (b.ii), and TNF-α (c.iii) concentration, and CCR2 (c.i.), CXCR4 (c.ii.) and PD-L1 (c.iii.) expression assessed. Data expressed as individual points, horizontal line represent median, box the interquartile range and whisker the range. Data analysed using multiple t-tests, only p<0.05 shown.

### Supplemental Figure 2: In response to a bacterial infection *in vitro*, patient lymphocyte display an immunosuppressive phenotype.

PBMCs were isolated from healthy volunteer (green) and patients who did not (blue) and did (red) develop a post-operative infection and the effect of stimulation with heat-killed bacteria (HKB) on CD4 (a.) and CD8 (b.) lymphocyte IL-2R (i.), CD28 (ii.), and IL-7R (iii.) expression, IL-2 (iv.) and IFN-y (v.) concentration, percentage of apoptotic cells (vi.), CTLA-4 (vii.) and PD-1 (viii.) expression and IL-10 (ix.) concentration assessed. Data expressed as individual points, horizontal line represent median, box the interquartile range and whisker the range. Data analysed using multiple t-tests, only p<0.05 shown.
